# Assessment of sacroiliitis using zero echo time magnetic resonance imaging: a comprehensive evaluation

**DOI:** 10.1007/s00247-025-06201-w

**Published:** 2025-02-25

**Authors:** Yunus Emre Bayrak, Törehan Özer, Yonca Anik, Sibel Balci, Duygu Aydin, Nihal Şahin, Hafize Emine Sönmez

**Affiliations:** 1 Kabaoğlu, Baki Komsuoğlu Boulevard, Kocaeli University Faculty of Medicine Department of Pediatrics, Division of Pediatric Rheumatology, Izmit, Kocaeli, 41001 Türkiye; 2 Department of Radiology, Kocaeli University, Kocaeli, Turkey; 3 Department of Biostatistics, Kocaeli University, Kocaeli, Turkey

**Keywords:** Low-dose computed tomography, Magnetic resonance imaging, Sacroiliitis, Zero echo time

## Abstract

**Background:**

Enthesitis-related arthritis (ERA), a subset of juvenile idiopathic arthritis (JIA), is characterized by frequent involvement of the sacroiliac (SI) joints.

**Objective:**

The aim of this study was to assess the effectiveness of zero echo time (ZTE) magnetic resonance imaging (MRI) in identifying structural lesions in patients with ERA. Conventional MRI pulse sequences often struggle to adequately visualize osseous and calcified tissues.

**Materials and methods:**

All MRI examinations were conducted using a 1.5-T (T) scanner. The MRI protocol included standard sequences such as fat-suppressed axial T2-weighted, axial T1-weighted, coronal short tau inversion recovery (STIR), and axial T2-weighted sequences. In addition to conventional MRI, a ZTE sequence was employed. Low-dose computed tomography (CT) served as the reference standard and was performed using a 640-multislice CT device. Structural lesions, including erosions, sclerosis, and changes in joint space, were compared between imaging modalities.

**Results:**

A total of 20 patients were included in the study (12 boys, 8 girls), with a median age at diagnosis of 14 years. ZTE-MRI demonstrated similar sensitivity to low-dose CT in detecting erosion (7 vs 8, *P* = 0.707). The interclass correlation coefficient (ICC) between low-dose CT and ZTE-MRI was 0.993 (*P* < 0.001), indicating excellent agreement. Moreover, ZTE-MRI showed strong agreement with low-dose CT in detecting sclerosis (ICC = 0.954, *P* < 0.001) and changes in joint space (ICC = 0.998, *P* < 0.001).

**Conclusions:**

Zero echo time imaging shows promise in providing sacroiliac joint visualization comparable to low-dose CT scans, thereby improving the detection of subtle erosion and sclerosis in these joints.

**Graphical Abstract:**

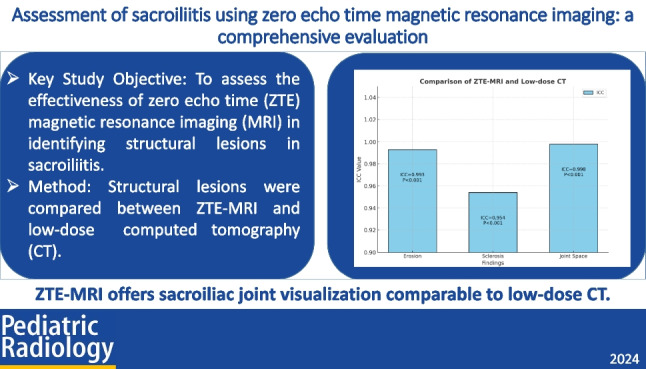

**Supplementary Information:**

The online version contains supplementary material available at 10.1007/s00247-025-06201-w.

## Introduction

Juvenile idiopathic arthritis (JIA) stands out as the leading cause of chronic arthritis in childhood, representing a broad range of diagnoses classified into seven distinct subgroups [1]. In our region, enthesitis-related arthritis (ERA), a subgroup of JIA, accounts for 18.9% of JIA cases, with a higher prevalence among boys aged 6 and older [2]. ERA predominantly affects the joints and entheses of the lower extremities, with potential progression to the spine or sacroiliac (SI) joints. It is distinguished by the absence of rheumatoid factor (RF) and is notably associated with human leukocyte antigen-B27 (HLA-B27) [2]. SI involvement represents a common manifestation of ERA, often presenting with lower back pain that is exacerbated during periods of inactivity. At the outset of the disease, approximately 30% of individuals with ERA exhibit symptomatic sacroiliitis, while hip involvement is noted in 10–15% of cases. Over time, these rates escalate, with sacroiliitis affecting 70% and hip arthritis involving 40% of individuals with ERA [3]. Given that sacroiliac joint involvement is linked to a poor prognosis, its thorough evaluation holds significant clinical importance [3]. Additionally, it is important to note that children can exhibit “silent sacroiliitis,” characterized by evidence of inflammation on imaging studies despite the absence of inflammatory back pain or clinical signs such as SI joint tenderness, pain on SI distraction maneuvers, or an abnormal Schober test. Hence, imaging methods play a crucial role in the assessment of these patients [4]. While magnetic resonance imaging (MRI) stands out as the most sensitive radiological method for detecting sacroiliitis, it poses challenges in identifying early erosive changes [3]. Conventional MRI pulse sequences are limited in their ability to reliably depict structures like osseous and calcified tissues. Recently, novel MRI sequences, such as zero–time echo (ZTE) MRI utilizing ultrashort echo times (UTEs), have emerged as a viable method to generate computed tomography (CT)-like images of ossified or calcified structures.

In this study, we aimed to assess the diagnostic accuracy of ZTE-MRI for detecting structural lesions in ERA patients, with low-dose CT serving as the reference standard.

## Material methods

This study is a cross-sectional observational study conducted between January 2024 and June 2024. The study included individuals diagnosed with ERA who were presented with sacroiliitis. Diagnosis of ERA was based on the classification criteria established by the International League of Associations for Rheumatology (ILAR) [1]. The required minimum sample size was calculated using priori-power analysis. The priori-power analysis with *α* (significance level) = 0.05 and 1–*β* (power) = 0.90 was conducted using PASS (Power Analysis and Sample Size) version 14.0 software to determine the required minimum sample size for an intraclass correlation coefficient (ICC) above 0.95 (almost perfect agreement). Since there was no similar study available in the literature to use as a reference, and no opportunity to conduct a pilot study, the priori-power analysis was performed to achieve an ICC value greater than 0.95, which corresponds to an “almost perfect agreement.” The minimum sample size was calculated as 14. To improve the power of our study, we decided to study with 20 patients. We also performed a post-hoc power analysis using our obtained results to determine the power of our study. The post-hoc power analysis was conducted using the ICC between CT and ZTE obtained for total score (ICC = 0.99), sample size *n* = 20, and significance level (*α*) = 0.05. The power of our study was calculated to be 96%.

The study meticulously documented demographic data, clinical manifestations, and laboratory findings (including white blood cell [WBC] count, erythrocyte sedimentation rate [ESR], C–reactive protein [CRP], and HLA-B27). It also included a comprehensive examination of treatment modalities.

The exclusion criteria comprised patients with psoriasis, familial Mediterranean fever, and inflammatory bowel disease.

Disease activity was assessed using the Juvenile Spondyloarthritis Disease Activity Index (JSpADA), a validated tool designed to measure disease activity specifically in juvenile spondyloarthritis [5].

The study adhered to the Helsinki Declaration guidelines for medical research involving human subjects and received approval from the local ethics committee. Written informed consents were obtained from patients and their parents.

### Magnetic resonance imaging procedure

All MRI procedures were performed by using a 1.5-T (T) scanner (GE Healthcare, Milwaukee, WI) with a 16-channel air coil. The MRI protocol includes a fat-suppressed axial T2-weighted sequence (TR/TE 2,500/85 ms, echo train length 16), axial T1-weighted sequence (TR/TE 610/minimum ms, echo train length 3), coronal T1-weighted sequence (TR/TE 590/minimum ms, echo train length 3), coronal short tau inversion recovery (STIR) sequence (TR/TE 4,250/42 ms, echo train length 16), and axial T2-weighted sequence (TR/TE 4,650/85 ms, echo train length 16). The imaging parameters included a 4-mm section thickness, a 1-mm intersection gap, a 384 × 384 matrix, and a 26 cm × 26 cm field of view. Additionally, a ZTE sequence (TR/TE 568/0 ms, 1.5-mm section thickness, no intersection gap, a 280 × 280 matrix, and a 40 cm × 40 cm field of view, flip angle 2°) was performed.

Active sacroiliitis was assessed by MRI according to the Assessment of Spondyloarthritis International Society (ASAS) criteria [6].

### Low-dose computed tomography protocol

To minimize temporal changes, low-dose CT and MRI were performed within 1 week. The CT scan was conducted using a 640-MSCT device, exposing patients to an average effective dose of 0.615 mSv per sacroiliac CT (Canon Medical Systems, Otowara, Japan). The scanning parameters included a voltage of 120 kV, automatically determined current based on the patient’s volume (80–95 mAs), a rotation time of 0.5 s, a layer thickness of 0.5 mm, a reconstruction interval of 2.5 mm, a window width of 2,700 Hounsfield units (HU), and a window level of 350 HU. No intravenous contrast agent was used.

### Comparison of the images

All MRI and CT scans were independently and anonymously evaluated by two pediatric radiologists, T.O., with 10 years of experience, and Y.A., with 25 years of experience, who were blinded to the patients’ information. All scores were conducted by two independent observers. ICC was used to determine the agreement between them. All imaging studies were independently evaluated by both readers following the same sequence and order. The assessments were conducted in the following order: first, conventional MRI; then, ZTE; and finally, low-dose CT. All images were anonymized and reviewed twice by each reader at separate time points, with the patient order randomized for each evaluation. A 2-day interval was maintained between the first and second assessments for each modality to minimize the likelihood of readers recalling the images or scores of the same patient. Structural lesions (erosion, sclerosis, joint space changes) were scored in the coronal plane. The joint was divided into anterior, middle, and posterior thirds [7]. From each third, the sections with the most intense structural changes were selected, ensuring that the selected sections were not consecutive or adjacent. While our scoring was based on a single section, we conducted a comprehensive evaluation of all patients and all sequences. Sclerosis was defined as the presence of sclerosis exceeding 5 mm in the subchondral area.

### Statistical analysis

All statistical analyses were performed using IBM SPSS for Windows version 29.0 (IBM Corp., Armonk, New York, NY). Shapiro–Wilk’s test was used to assess the normality assumption. Since the normality assumption did not hold, continuous variables were presented with median and interquartile range (IQR). Categorical variables were presented with the number of observations and percentages. Friedman’s two-way analysis of variance (ANOVA) was conducted for dependent group comparisons. Dunn’s test was used for the pairwise multiple comparisons. Using low-dose CT as the reference standard, the agreement between ZTE and low-dose CT was determined by ICC. These parameters were assessed at the quadrant level for erosion and sclerosis, and at the joint level for joint space changes (Supplementary Material 1). A *P*-value < 0.05 was considered statistically significant.

## Results

### Demographic, clinical, and laboratory characteristics of the study group

A total of 20 patients with ERA were evaluated. Of them, 8 (40%) were female. The median current age, age of diagnosis, and follow-up period were 14 (11–15) years, 10 (8–14) years, and 17 (7–32) months, respectively. Among 20 patients, 16 (80%) had a family history of rheumatic disease, and in 1 (5%), consanguinity marriage was reported.

At time of the diagnosis, the most common symptom was low-back pain in 19 (95%), followed by morning stiffness in 16 (80%), peripheral arthritis in 15 (75%), heel pain in 9 (45%), and enthesitis in 8 (40%) patients. None of the patients had uveitis. HLA-B27 was positive in 18 (90%) patients. The median WBC count was 7,363 (6,365–9,647) mm^3^, hemoglobin was 12.1 (11.25–12.87) mg/dL, platelet count was 311,500 (270,000–363,000) mm^3^, CRP was 3.78 (0.13–10.01) mg/dL, and ESR was 17.5 (8.75–40.25) mm/h at diagnosis. The median JSpADA was 4 (2.62–4.37) at diagnosis. All patients initially received non-steroidal anti-inflammatory drugs (NSAIDs). Of these, 17 (or 85%) also used disease-modifying antirheumatic drugs (DMARDs), which for 14 patients included methotrexate and for 3 patients included sulfasalazine. Additionally, 10 (or 50%) of the patients were treated with biological agents, including 8 patients with etanercept and 2 with adalimumab.

At the time of radiologic evaluation, the median (IQR) counts of active joints and enthesitis were 2 (1–3.75) and 0 (0–0), respectively. Fourteen patients (70%) exhibited clinical sacroiliitis, and 9 patients (45%) experienced morning stiffness. The median JSpADA was 2.75 (2–3.37). The median CRP level was 2.53 (0.65–9.33) mg/dL, and ESR was 14 (4.75–19) mm/h. Seven patients were receiving DMARDs with a median duration of 6 months, and 11 patients were receiving biological agents with a median duration of 6 months.

### Comparison of images

**Fig. 1:**
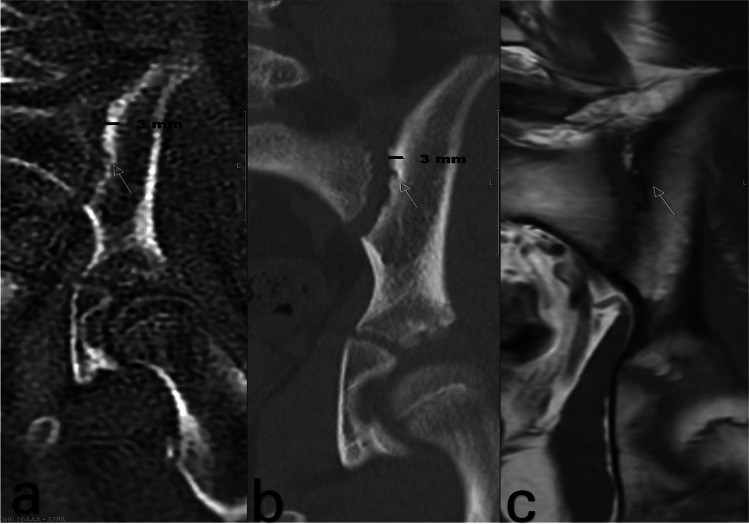
A 16-year-old boy with sacroiliitis. **a** Coronal ZTE-MRI shows millimetric cortical erosion in the left iliac wing (*arrow*) and increased density and signal thickness caused by subcortical sclerosis (*black straight lines*).** b** Coronal low-dose CT image shows millimetric cortical erosion in the left iliac wing (*arrow*) and increased density and signal thickness caused by subcortical sclerosis (*black straight lines*).** c** Coronal T1-weighted image shows millimetric cortical erosion in the left iliac wing (*arrow*) and subcortical sclerosis are not distinguishable. *CT*, computed tomography; *ZTE-MRI*, zero–time echo magnetic resonance imaging

**Fig. 2:**
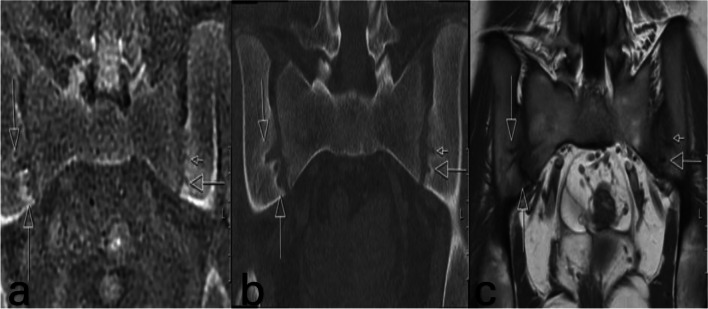
A 16-year-old girl with sacroiliitis. **a** Coronal ZTE-MRI shows cortical erosion areas (*arrow*). **b** Coronal low-dose CT image shows cortical erosion areas (*arrow*). **c** Coronal T1-weighted image indicates areas of cortical erosion (*arrow*), but these appear smaller. *CT*, computed tomography; *ZTE-MRI*, zero–time echo magnetic resonance imaging

The ICC values ranged from 0.93 to 0.99 (low-dose CT, 0.99; ZTE-MRI, 0.98; conventional MRI, 0.93), indicating excellent inter-observer reliability. The diagnostic accuracy of ZTE-MRI for detecting erosion, sclerosis, and joint space changes was evaluated. ZTE-MRI showed comparable sensitivity in detecting erosions on the quadrant level. The median total score of erosion was 8 (1.25–14.5) in low-dose CT and 7 (1–13) in ZTE-MRI (*P* = 0.707). However, the median total score of erosion was 0.5 (0–6.25) in conventional MRI (*P* < 0.001). The ICC between low-dose CT and ZTE-MRI was 0.993 (*P* < 0.001) while conventional MRI showed low agreement with low-dose CT (ICC = 0.679, *P* < 0.001). When we evaluated iliac and sacral erosion separately, the median total score of iliac erosion was 2.5 (0.25–11.75) in low-dose CT and 2.5 (0.25–11.75) in ZTE-MRI (*P* = 1) (ICC = 0.996, *P* < 0.001). The median total score of sacral erosion was 2 (0–7.75) in low-dose CT and 2 (0–6.75) in ZTE-MRI (*P* = 1) (ICC = 0.930, *P* < 0.001) (Figs. 1 and 2).

**Fig. 3:**
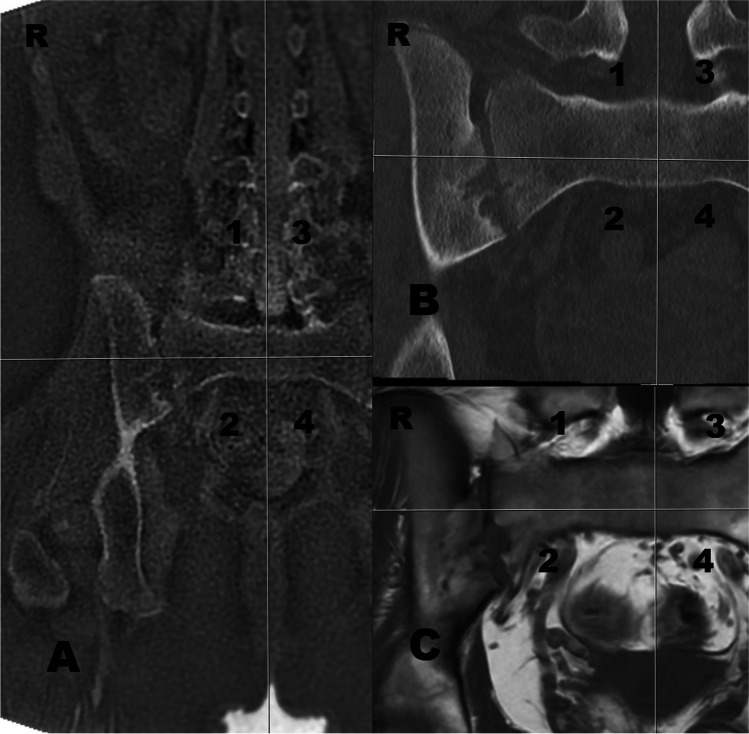
A 16-year-old girl with sacroiliitis. **a** Coronal ZTE-MRI received a score of 0 in the 1st segment and a score of 2 (pseudowidening) in the 2nd segment. **b** Coronal low-dose CT received a score of 0 in the 1st segment and a score of 2 (pseudowidening) in the 2nd segment. **c** Coronal T1-weighted image received a score of 0 in the 1st segment and a score of 2 (pseudowidening) in the 2nd segment. *CT*, computed tomography; *ZTE-MRI*, zero–time echo magnetic resonance imaging

The median total sclerosis score was 0 (0–1) in low-dose CT and 0 (0–1) in ZTE-MRI (*P* = 1). ZTE-MRI showed a strong agreement with low-dose CT in terms of detecting sclerosis (ICC = 0.954, *P* < 0.001). When we evaluated iliac and sacral sclerosis separately, the median total score of iliac sclerosis was 0 (0–1) in low-dose CT and 0 (0–1) in ZTE-MRI (*P* = 1). The median total score of sacral sclerosis was 0 (0–0) in low-dose CT and 0 (0–0) in ZTE-MRI (*P* = 1) (Figs. 1, 2, and 3).

The median score of joint space changes was 3.5 (0–9.75) in low-dose CT and 3 (0–9) in ZTE-MRI (*P* = 1). ZTE-MRI revealed a strong agreement with low-dose CT in terms of detecting joint space changes (ICC = 0.998, *P* < 0.001) (Table 1).

**Table 1:** The diagnostic accuracy of ZTE-MRI and low-dose CT

	ZTE-MRI vs MRI		Low-dose CT vs MRI		ZTE-MRI vs low-dose CT	
	ICC	*P*-value	ICC	*P*-value	ICC	*P-*value
Erosions	0.710	< 0.001	0.679	< 0.001	0.993	< 0.001
Sclerosis	0.954	< 0.001	0.954	< 0.001	1	-
Joint space changes	0.947	< 0.001	0.937	< 0.001	0.998	< 0.001

*CT*, computed tomography; *ICC*, intraclass correlation coefficient; *MRI*, magnetic resonance imaging; *ZTE*, zero echo time

## Discussion

Using low-dose CT as the reference standard, we found that ZTE-MRI demonstrated superior accuracy compared to conventional MRI in detecting erosion, sclerosis, and changes in joint space. Specifically, ZTE-MRI showed a strong agreement with low-dose CT in visualizing erosions, sclerosis, and joint space changes. This study marks the pioneering use of ZTE-MRI in pediatric rheumatic diseases.

While MRI is widely regarded as the most sensitive standard imaging modality for musculoskeletal conditions, conventional MRI pulse sequences face limitations in accurately depicting structures like osseous and calcified tissues with ultrashort (< 1 ms) T2 relaxation times, primarily due to constraints on achieving minimal echo times. Specific MRI sequences have enabled the use of ultrashort echo times in the context of musculoskeletal imaging. ZTE-MRI, a recent MRI modality, yields CT-like images of ossified or calcified structures [8]. In ZTE-MRI, signal acquisition begins almost immediately after the radiofrequency pulse is applied, effectively making the echo time zero. This minimizes signal loss from tissues with very short T2 relaxation times. ZTE-MRI began to be utilized for rheumatic diseases in the adult population. Li et al. [9] evaluated the diagnostic performance of ZTE-MRI in detecting structural lesions in the sacroiliac joints, using CT as the reference standard. They found that ZTE-MRI revealed superior diagnostic performance in detecting structural lesions compared to routine T1-weighted MRI, with reliability comparable to CT. Most recently, Lin et al. [7] showed that ZTE-MRI could improve the diagnostic accuracy of detecting erosions and sclerosis of the sacroiliac joint in patients suspected of having axial spondyloarthritis (axSpA). The use of ZTE-MRI in pediatrics is limited. Sandberg et al. [10] evaluated the consistency of ZTE-MRI and CT in measuring cortical thickness in different joint regions in 20 pediatric patients and they found that cortical bone visualization on ZTE-MRI is diagnostic and is comparable to conventional CT. However, there are no studies evaluating the sacroiliac joint using ZTE-MRI in children. Our study showed that ZTE-MRI demonstrated superior accuracy compared to conventional MRI in detecting erosion, sclerosis, and joint space changes with a strong agreement with low-dose CT. However, it is likely that sclerosis formation is a more chronic and prolonged process compared to erosion formation. Therefore, sclerosis was not observed with the same prevalence as seen in adults within the pediatric patient group. Furthermore, we applied a 5-mm cut-off for sclerosis. The absence of a validated threshold for identifying sclerosis may also have been impactful.

Studies in adults have demonstrated that structural damage in the sacroiliac joint significantly impacts the functional abilities and spinal mobility of patients with axial spondyloarthritis [11]. Despite limited studies in childhood, reports have indicated a deleterious effect of sacroiliac involvement on the course of JIA. For instance, Chan et al. [12] demonstrated that an extended duration from initial symptom onset to diagnosis was associated with structural changes in the sacroiliac joint in ERA patients and they underscored the significance of timely diagnosis and early initiation of treatment. Furthermore, Rumsey et al. [13] demonstrated that the presence of sacroiliitis increased the need for biologic treatment. Therefore, early detection of damage to the sacroiliac joint is crucial for the timely initiation of effective treatments and for preventing the progression of the damage.

The significant advantages of ZTE-MRI are that it extends the MRI time by only a few minutes and, unlike tomography, does not expose children to harmful radiation. Our study was a preliminary study in terms of adapting new MRI techniques to childhood diseases. The primary limitation of our study was the absence of healthy controls, which was unavoidable due to ethical concerns regarding the potential ionizing radiation from CT scans. Another notable limitation was the single-center design. In future research, we aim to validate our findings in a multicenter cohort to enhance their accuracy and reliability.

In conclusion, ZTE-MRI is a feasible technique that provides diagnostic–quality images in children with sacroiliitis. Future studies with a multicenter design focusing on sacroiliitis may improve the clinical acceptance of ZTE.

## Supplementary Information

Below is the link to the electronic supplementary material.

Supplementary file1 (PDF 297 KB)

## Data Availability

No datasets were generated or analysed during the current study.
