# Supplementary material for: Assessment of sacroiliitis using zero echo time magnetic resonance imaging: a comprehensive evaluation
Source: Pediatr Radiol. 2025 Feb 25;55(5):999–1005. doi: 10.1007/s00247-025-06201-w (PMC12065742; doi:10.1007/s00247-025-06201-w)
Supplement: Supplementary file 1 — Supplementary file1 (PDF 297 KB) [file 247_2025_6201_MOESM1_ESM.pdf]

Supplementary material 1. Scoring method for structural abnormalities of the sacroiliac joint

	Erosion	Sclerosis	Joint space changes
0	No erosion	No sclerosis or limited sclerosis (less than 5 mm)	No joint space changes
1	Small isolated erosions (1–2)	Evident sclerosis (≥5 mm)	Questionable widening or narrowing
2	Definite erosions (3–5; <3 mm) or larger single erosion (>3 mm)		Pseudowidening
3	Multiple (>5) or confluent erosions		Partial ankylosis
4			Extensive/total ankylosis

[a]These parameters were assessed at the quadrant level for erosions and sclerosis, and at the joint level for joint space changes [7].

Supplementary figure 1. Segmentation on coronal images. For segmentation, in coronal sections, the slices where the sacral foramina first appeared within the field of view, along with the slices anterior to them, were defined as the anterior 1/3. The slices where the neural foramina were visible in a circular manner along with the sacral bones were defined as the middle 1/3. The slices posterior to these were defined as the posterior 1/3. Additionally, all segments were further divided into two equal parts: superior and inferior. Thus, 24 segments were obtained for the evaluation of erosion and sclerosis, and 12 segments for the evaluation of joint space for each patient.

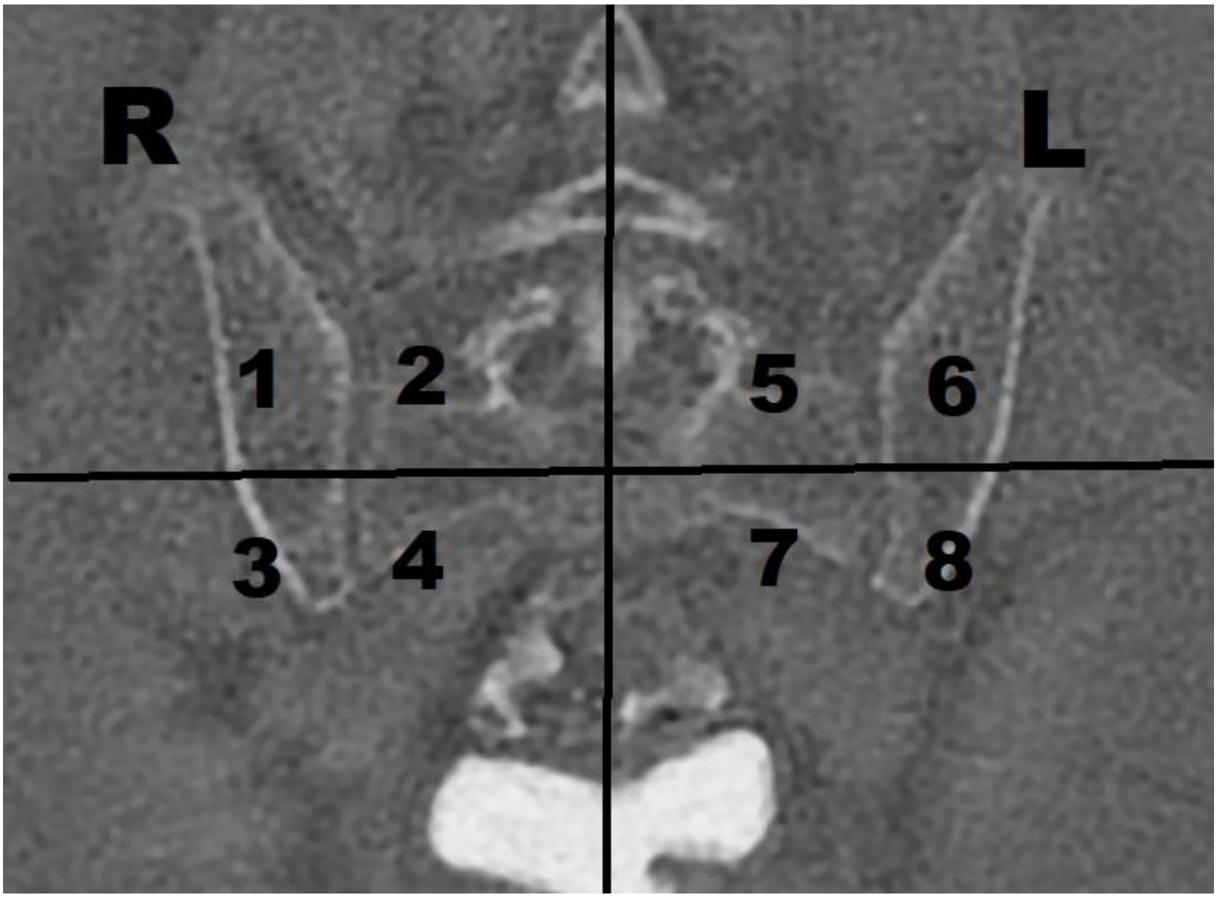
R
L
1
2
5
6
3
4
7
8

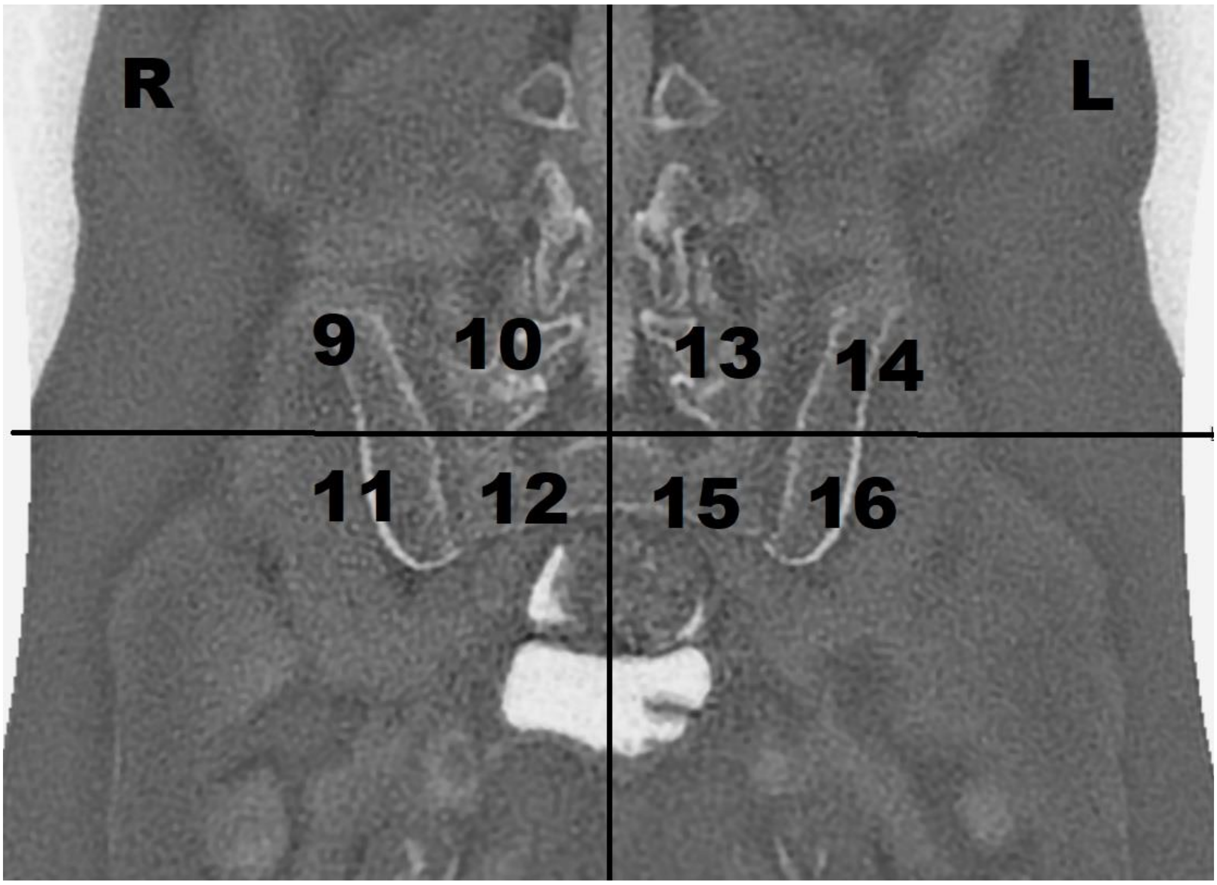
R
L
9
10
13
14
11
12
15
16

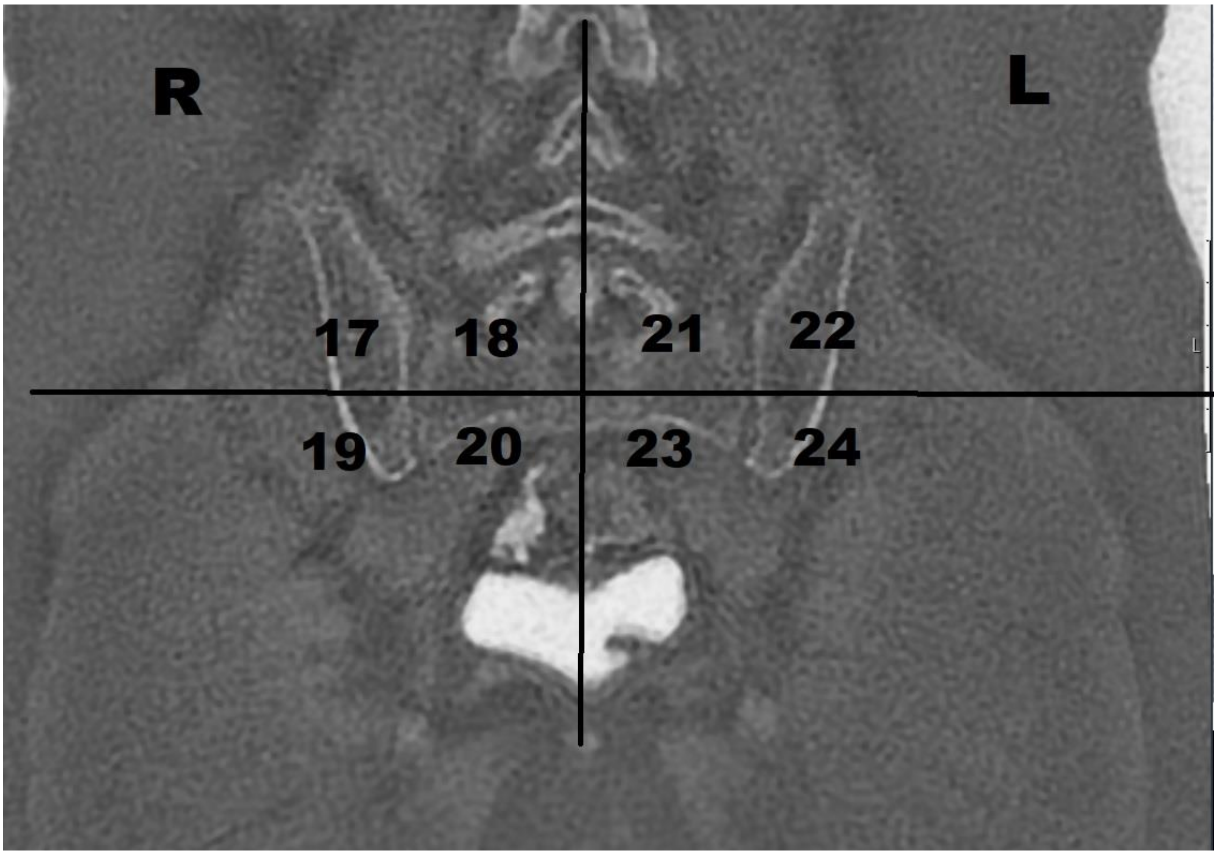
R
L
17
18
21
22
19
20
23
24
